# *Immortal* Ring-Opening Polymerization of *rac*-Lactide Using Polymeric Alcohol as Initiator to Prepare Graft Copolymer

**DOI:** 10.3390/polym8010017

**Published:** 2016-01-15

**Authors:** Na Liu, Bo Liu, Changguang Yao, Dongmei Cui

**Affiliations:** 1 State Key Laboratory of Polymer Physics and Chemistry, Changchun Institute of Applied Chemistry, Chinese Academy of Sciences, Changchun 130022, China; liunn@ciac.ac.cn (N.L.); liubo@ciac.ac.cn (B.L.); chgyao@ciac.ac.cn (C.Y.); 2 University of the Chinese Academy of Sciences, Changchun Branch, Changchun 130022, China

**Keywords:** *immortal* ring-opening polymerization, RAFT polymerization, polymeric alcohol, grafting-from, nanoporous film

## Abstract

In the presence of a small molecular protic initiator, *immortal* ring-opening polymerization (ROP) of lactide (LA) is a highly efficient strategy to synthesize polylactide in a controllable manner, while using polymeric alcohol as an initiator has been less investigated. A series of polymeric alcohols (PS–OH) composed of styrene and 4.3%–18% hydroxyl functional styrene (diethyl(hydroxy(4-vinylphenyl)methyl)phosphonate, St–OH) were synthesized through reversible addition-fragmentation transfer (RAFT) polymerization. Using PS–OH as an initiator, the *immortal* ROP of *rac*-LA was catalyzed by dibutylmagnesium (Mg*^n^*Bu_2_) under various ratios of monomer to hydroxyl group within PS–OH to generate polystyrene-*g*-polylactide (PS–*g*–PLA) copolymers with different graft lengths. After thermal annealing at 115 °C, the PLA domain aggregated to nanospheres among the PS continuum. The size of the nanospheres, varying from 130.1 to 224.2 nm, was related to the graft density and length of PS–*g*–PLA. Nanoporous films were afforded through chemical etching of the PLA component.

## 1. Introduction

Graft copolymers are an important building blocks to form self-assembly aggregates because of convenient adjusting of the self-assembly behavior by changing the grafting side-chain density and length [1,2,3,4,5,6,7,8] as compared with the corresponding widely investigated block copolymers. Graft copolymers could be synthesized by three methods of grafting through [9,10,11,12,13], grafting from [14,15] and grafting on [16] by merit of the anionic/radical living polymerization techniques, of which the grafting-from is considered a particularly attractive procedure [17] and a large number of graft copolymers with various backbones and side-chains have been synthesized by combining the ‘‘grafting-from’’ strategy with living/controlled radical polymerization. In comparison, the combination of ring-opening coordination polymerization of cyclic ester and the grafting-from strategy to synthesize a graft copolymer has aroused less attention [18] and few successful examples, since coordination polymerization is usually non-living without functional group fidelity as the further building block for grafting joints.

Biodegradable and biocompatible polylactide (PLA) derives from the bio-resourced lactic acid and has become one of the most promising environmentally friendly materials. Up until now, the highly efficient strategy of preparing PLA has been no doubt attributed to the *immortal* ring-opening polymerization (ROP) of lactide (LA) coined by Inoue *et al.* [19,20,21] when they studied the ROP of epoxides using a metal-based catalyst under the presence of excess amounts of alcohol. Therefore, *immortal* polymerization allows us to generate hundreds of polymer chains per metal with precisely controlled molecular weight and molecular weight distribution, moreover with functional group ends. To date, the most suitable and widely investigated protic initiators are small molecular alcohols (ROH) to give hydroxyl end-functionalized PLA [22,23,24,25,26,27,28] or star PLA [29]. The behavior of using polymeric alcohols as the protic initiators has been explored less.

Herein we report firstly the synthesis of a series of polymeric alcohols (PS–OH) composed of styrene and 4.3%–18.0% St–OH through reversible addition-fragmentation transfer (RAFT) polymerization, and the *immortal* ROP of *rac*-LA catalyzed by Mg*^n^*Bu_2_ with PS–OH as the initiator to generate polystyrene–*g*–polylactide (PS–*g*–PLA) copolymers with different graft lengths. The relationship of the thermal aggregation behavior of the (PS–*g*–PLA) graft copolymers and the formation of the nanoporous microstructure membranes will also be discussed.

## 2. Experimental Section

### 2.1. General Procedures

All reactions were carried out under a dry nitrogen atmosphere using Schlenk techniques or in a glovebox filled with dry nitrogen. THF was dried by distillation over sodium with benzophenone as indicator under a nitrogen atmosphere and was stored over freshly cut sodium in a glovebox. Mg*^n^*Bu_2_, azodiisobutyronitrile (AIBN) and dibenzyl carbonotrithioate were purchased from Sigma-Aldrich (St. Louis, MO, USA). *rac*-LA was recrystallized with dry ethyl acetate three times. Styrene was dried over CaH_2_ under stirring for 48 h and distilled under reduced pressure before use. Glassware and flasks using in the polymerization were dried in an oven at 115 °C overnight and exposed to a vacuum-nitrogen cycle three times. The molecular weight and weight distribution of the polymers were measured using a TOSOH HLC 8220 GPC instrument at 40 °C with THF as eluent against polystyrene standards. Polymers for NMR spectroscopy measurements were prepared by use of NMR tube sealed by paraffin film. The ^1^H and ^13^C NMR spectra were recorded on a Bruker AV400 spectrometer (FT, 400 MHz for ^1^H, 100 MHz for ^13^C, Brucker BioSpin GmbH, Rheinstetten, Germany). AFM was in tapping mode under an air atmosphere with SPA380HV with an SPI3800N controller (Seiko Instruments Inc., Chiba, Japan). The piezo scanner was able to scan with a horizontal range of 150 μm and a vertical range of 150 μm. TEM was carried out on a JEM-1011 (JEOL Ltd, Tokyo, Japan) device. The electron gun was equipped with a field emission electron source and was operated at 100 keV.

### 2.2. Material and Methods

#### 2.2.1. A Typical Procedure to Synthesize PS–OH

A typical polymerization procedure was described here. In a glovebox, AIBN (10 μmol, 1.6 mg), dibenzyl carbonotrithioate (100 μmol, 29.1 mg), St–OH [30,31] (1 mmol, 0.27 g) and styrene (10 mmol, 1.04 g) were added into a 30 mL ampule. Then, the ampule was quickly taken out of the glovebox and put into a pre-equilibrated 70 °C oil bath. After 24 h, the product was precipitated in excess ethanol and dried *in vacuo* at 40 °C for constant weight. Finally, a yellow powder PS–OH was obtained. The molecular weight and the molecular weight distribution of the resulting polymer were characterized by GPC analysis. The content of St–OH was determined by ^1^H NMR.

#### 2.2.2. A Typical Procedure for Immortal ROP

A typical polymerization of *rac*-LA was performed in an ampule (30 mL) in a glovebox. The polymeric initiator PS_8.6_–OH (1.18 g) was completely resolved by THF (20 mL). Then, Mg*^n^*Bu_2_ (43 μL, 1 M) was injected into the system. After 10 min, *rac*-LA (4.3 mmol, 0.62 g) was quickly added. The polymerization mixture was stirred at room temperature for 1 h. Several drops of acidified methanol were injected to terminate the polymerization. Then the reaction solution was poured into excess ethanol. After filtration, the residue was dried in vacuum at 40 °C for constant weight. The molecular weight and the molecular weight distribution of the resulting polymer were characterized by GPC analysis. The graft length was determined by ^1^H NMR.

#### 2.2.3. Substrate Preparation and Spin-Coating

The silicon wafers for AFM were pretreated in a 2:1 *v*/*v* solution of 98% H_2_SO_4_ and 30% H_2_O_2_ at 100 °C for 1 h to generate a SiO_χ_ layer on the surface. Then, the silicon wafers were cleaned by successive sonication steps in acetone and deionized water for 15 min each three times and blow-drying by nitrogen thoroughly before spin-coating. Thin films of the PS–*g*–PLA were prepared by spin-coating a chlorobenzene solution of PS-*g*-PLA (5 mg·mL^−1^) on silicon substrates first at 500 rpm for 10 s and at 3000 rpm for 30 s. The thin films were dried at 40 °C under a vacuum pressure. The preparation of the glass sheets was similar to the procedure of the silicon wafers except for acid picking procedure.

#### 2.2.4. Thermal Annealing

The thermal annealing process was performed at 115 °C for 4 h and then gradually cooled to room temperature.

#### 2.2.5. Etching Procedure of the PS–*g*–PLA

PS–*g*–PLA film was placed 9 cm from the lamps and exposed to UV chamber (An-Ting Electronic Instrument Factory, Shanghai, China) (wavelength 365 nm, power 16 W) for 1 h under air, then dipped into a 0.5 M sodium hydroxide solution containing 40/60 (*v*/*v*) methanol/water. After 30 min, the PS–*g*–PLA film was washed with a 40/60 (*v*/*v*) methanol/water and collected on carbon-coated copper TEM grid.

## 3. Results and Discussions

### 3.1. Synthesis of Polymeric Initiator (PS–OH)

**Scheme 1:**
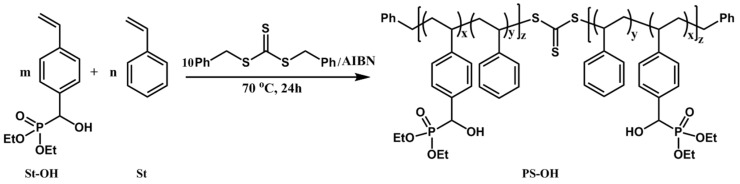
Synthesis of PS–OH by RAFT copolymerization of styrene and St–OH.

**Figure 1:**
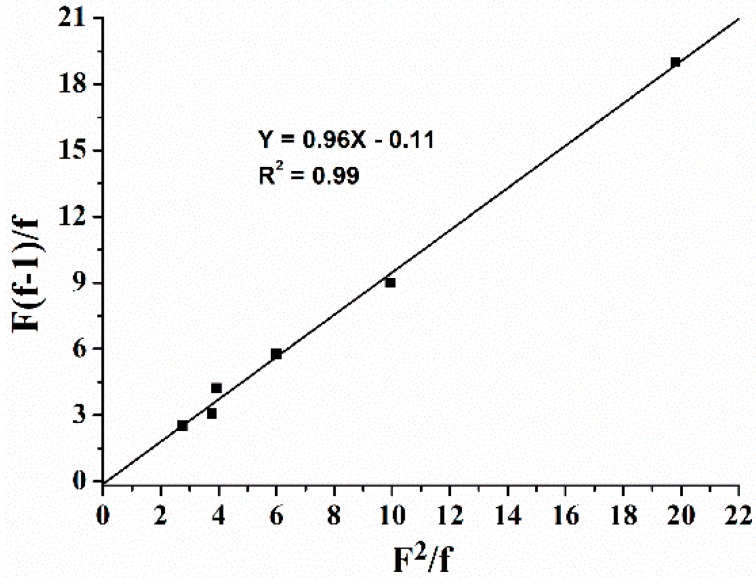
Fineman-Ross plot for copolymerization of styrene and St–OH at 70 °C (F = [St]/[St–OH] in feed, f = [St]/[St–OH] in copolymer, *r*_St_ = 0.96, *r*_St–OH_ = 0.11).

The polymeric initiator composed of styrene and 4.3%–18% St-OH was synthesized in bulky via RAFT polymerization using AIBN and dibenzyl carbonotrithioate as the initiators at 70 °C (Scheme 1). Firstly, copolymerization of styrene and St–OH was performed under various St–OH-to-St ratios to low conversions. The reactivity ratios of *r*_St_ = 0.96 and *r*_St–OH_ = 0.11 were determined according to the Finemann-Ross equation (Figure 1) with *r*_St_ < 1, *r*_St–OH_ < 1, and *r*_St_ × *r*_St–OH_ < 1, suggesting the azeotropic point appeared at the St–OH-to-St feeding ratio of 0.04:1, where the composition of the copolymer was kept constant at various monomer conversions [32]. Based on this, we chose St–OH-to-St feeding ratios varying from 0.05:1 to 0.25:1 to synthesize the statistical copolymers PS–OH with different OH contents (Table 1). The resonance of the methine proton within St–OH shifts upfield (4.90 ppm) (Figure S1, Supplementary Material) compared to that (5.00 ppm) (Figure S2, Supplementary Material) in the monomer. The St–OH content within the copolymer was calculated on the basis of the relative integration intensity between the aromatic protons at 6.50 ppm and the methine proton of St–OH. Therefore, all polymerizations were carried out at 70 °C for 24 h to total monomer conversions around 70%. When the St–OH:St feed ratio (0.5:10 mmol) was near the azeotropic point, the St–OH content within the copolymer was 4.3%, which is close to that in the feeding [32]. Increasing the amount of St–OH from 1.0 to 2.5 mmol, copolymers with St–OH content increasing from 8.9% to 18.0% were obtained, although the St–OH contents were slightly lower than those loading amounts owing to the relatively low competitive reactivity of monomer St–OH (Table 1, entries 1–4).

**Table 1:** Syntheses of PS-OH by RAFT copolymerization of styrene and St–OH. ^a^

Entry	St–OH (mmol)	St (mmol)	Yield ^b^ (%)	Cont._theory_^c^ (%)	Cont. ^d^ (%)	*M*_n_^e^ (×10^4^)	PDI ^e^
1	0.5	10	68.9	4.8	4.3	0.82	1.17
2	1.0	10	71.3	9.1	8.9	0.88	1.14
3	1.5	10	69.3	13.0	12.5	0.90	1.16
4	2.5	10	74.4	20.0	18.0	0.95	1.18

^a^ Conditions: AIBN: 10 μmol, dibenzyl carbonotrithioate: 100 μmol, *T*_p_ = 70 °C, Time = 24 h; ^b^ Isolated yield after precipitation; ^c^ Cont._theory_ = *n*_St–OH_/(*n*_St_ + *n*_St–OH_); ^d^ Determined by ^1^H NMR spectroscopy; ^e^ Determined by size exclusion chromatography calibrated *vs.* polystyrene standards.

### 3.2. Immortal ROP Using PS–OH as Initiator to Prepare PS–g–PLA

**Figure 2:**
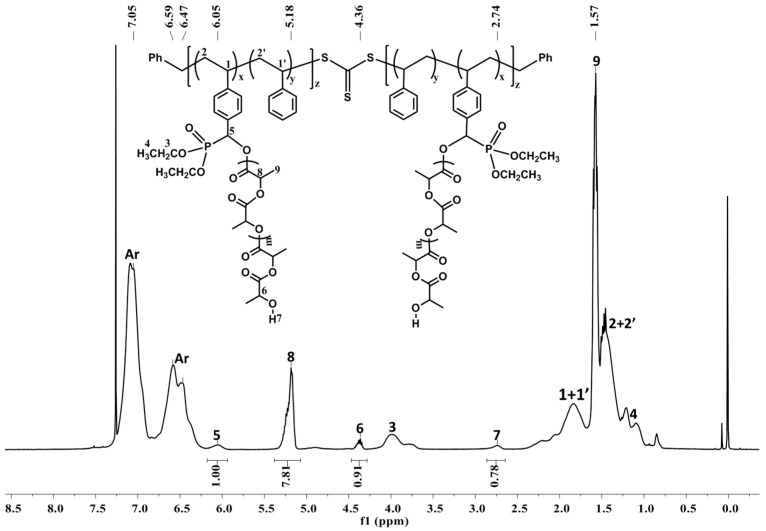
The ^1^H NMR spectrum (400 MHz, CDCl_3_, 298 K) of the resulting PS_8.6_–*g*–PLA_4.8_ (*M*_n,SEC_ = 10,600 g/mol, PDI = 1.44, Table 2, entry 5).

Recently, we reported that the ligand-free, simple, and readily available Mg^n^Bu_2_ and Ph_2_CHOH can mediate the rapid *immortal* ROP of L-LA with high catalyst activity and remarkable catalyst efficiency to generate up to 500 PLA polymer chains per metal center, in which the molecular weights were accurately controlled by the [Ph_2_CHOH]_0_ loading relative to [Mg]_0_, while the molecular weight distributions remained narrow and nearly constant [25]. Hence, the hydroxyl functionalized polystyrenes (PS–OH) obtained above were anticipated to play the role of Ph_2_CHOH as the chain transfer agent. The binary catalytic system composed of Mg^n^Bu_2_ and an excess amount of PS-OH was assayed in the activity toward the *immortal* ROP of *rac*-LA. The use of *rac*-LA to give atactic PLA was important to the degradation of PLA [33]. Firstly, the polymeric initiator PS_4.3_–OH (1.11 g contains 0.43 mmol St–OH and the footnote represented the content of St–OH within PS–OH) was dissolved in THF, then hexane solution of Mg^n^Bu_2_ (43 μL, 1 M) was injected. After 10 min, *rac*-LA (0.62 g, 4.3 mmol) was transferred into the above solution, which was kept stirring at r.t. for 1 h to achieve quantitative conversion (Table 2, entry 1). In the ^1^H NMR of the resultant polymer PS–*g*–PLA (Figure 2), the resonance of the methine proton within St–OH appears at 6.05 ppm (H5), shifting downfield compared to that within PS–OH (4.90 ppm), which is similar to that within the phosphonate ester attaching to polylactide [30] (6.14 ppm), indicating that the PLA chains indeed grafted from the hydroxyl groups of PS–OH. The multiplets at 4.36 ppm (H8) are assigned to the methine protons of LA connected with the hydroxyl group (H7, 2.74 ppm) [25]. The multiplets at 5.18 and 1.57 ppm are attributed to the methine and methyl protons of the PLA polymer, respectively. The graft length of the LA side-chain was 10.2, calculated according to the following formula: *G*_N_ = (*I*_5.18_ + *I*_4.36_)/(2 × *I*_6.05_) (Table 2, entry 1), close to the theoretical graft value of 10. Fixing the ratio of [LA]_0_:[St–OH]_0_ to be 100:10, no matter how the content of St–OH within PS–OH (5% to 20%) was changed, the grafted PLA chain length in the obtained PS–*g*–PLA copolymers was maintained around the designed 10 (Table 2, entries 1–4). Hence, a series of PS–*g*–PLA samples with the same graft length and different graft densities were prepared. Similarly, keeping the content of St–OH constant (8.6%), the change of the ratio of [LA]_0_:[St–OH]_0_ in the range of 5:1 to 20:1 caused the grafted PLA chain length to increase from 4.8 to 19.8 (Table 2, entries 2, 5 and 6), indicating that the grafted side-chain length could be swiftly controlled.

**Table 2:** Polymerization of *rac*-LA catalyzed by Mg^n^Bu_2_/PS–OH. ^a^

Entry	PS–OH/g	*rac*-LA (mmol)	Graft length (G_N_) ^b^	*M*_n_^c^ (×10^4^)	PDI ^c^
1	PS_4.3_–OH/1.11	4.3	10.2	1.47	1.42
2	PS_8.6_–OH/1.18	8.6	9.8	1.61	1.45
3	PS_12.5_–OH/1.24	12.5	10.1	1.73	1.55
4	PS_18.0_–OH/1.34	18.0	9.7	1.81	1.48
5	PS_8.6_–OH/1.18	4.3	4.8	1.06	1.44
6	PS_8.6_–OH/1.18	17.2	19.8	1.52	1.51

^a^ [Mg^n^Bu_2_]_0_:[rac-LA]_0_ = 1:100, THF: 20 mL, Temperature: 25 °C, Time: 1 h; ^b^ Determined by ^1^H NMR spectroscopy; ^c^ Determined by size exclusion chromatography calibrated *vs.* polystyrene standards.

### 3.3. Annealing-Mediated Assembly of the PS–g–PLA Films

**Figure 3:**
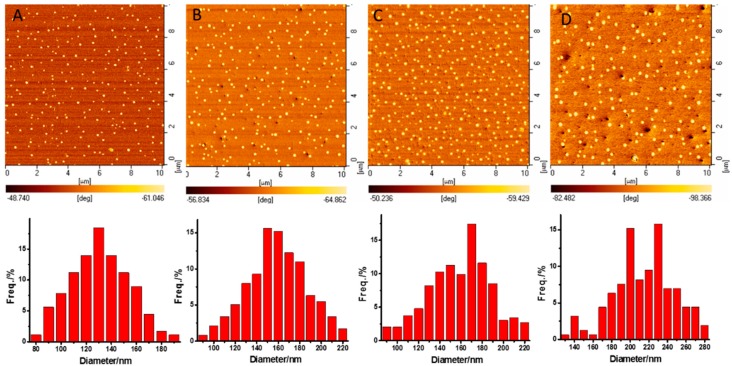
AFM phase images and the sizes of the nanospheres of the PS–*g*–PLA with different graft densities after thermal annealing at 115 °C for 4 h: (**A**) PS_4.3_–*g*–PLA_10_; (**B**) PS_8.6_–*g*–PLA_10_; (**C**) PS_12.5_–*g*–PLA_10_; (**D**) PS_18.0_–*g*–PLA_10_.

**Figure 4:**
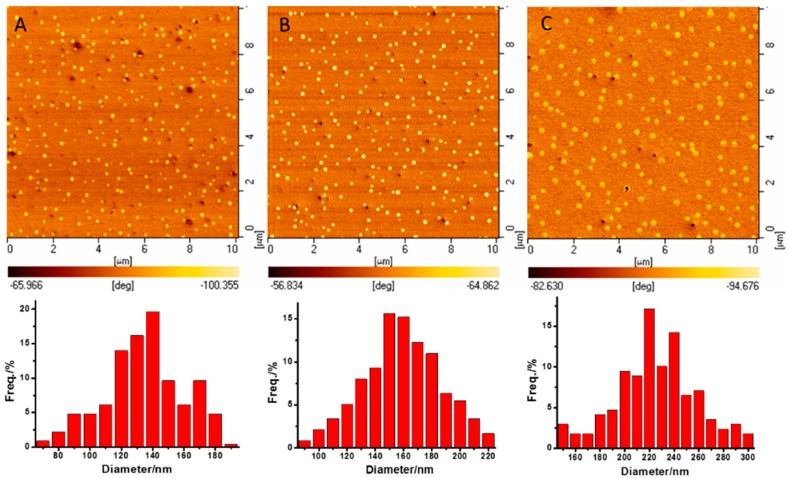
AFM phase images of the PS–*g*–PLA with different graft lengths after thermal annealing at 115 °C for 4 h: (**A**) PS_8.6_–*g*–PLA_5_; (**B**) PS_8.6_–*g*–PLA_10_; (**C**) PS_8.6_–*g*–PLA_20_.

The chemical incompatibility between the PS and PLA graft enabled the PS–*g*–PLA films to spontaneously form microphase-separation structures with the lowest free energy. However, it was difficult to self-assemble with reliance only on the inner superiority of mutual balance between the chemical incompatibility and covalent bond linkage of the constituent graft copolymer. The thermal field which provides energy for the thermodynamic movement of PS and PLA chains, and thus promotes the reorganization of their conformation and orientation [34], is thought to be a facile way to regulate the self-assembling of PS–*b*–PLA films [35]. The segment adjustment was achieved only when the system temperature was higher than the *T*_g_ of both PS (109.5 °C) and PLA (69.65 °C). Therefore, the thermal annealing of the PS–*g*–PLA film was carried out at 115 °C for 4 h. The phase images for PS–*g*–PLA showed a dispersive nanosphere phase for PLA and a continuous phase structure for PS. The average diameter (D) and the density of the nanosphere within PS_4.3_–*g*–PLA_10_ (4.3 represents the graft density (4.3%), 10 represents the length of PLA), measured using Nano Measurer 1.2, were 130.1 nm and 179/(100 μm^2^), respectively (Figure 3A; Table 3, entry 1). Keeping the length of the PLA graft constant, increasing the graft density from 8.6% to 12.5%, the density of the nanospheres increased correspondingly from 239 to 293/(100 μm^2^), while the average diameters were nearly similar (158.2 and 152.4 nm) (Figure 3B,C; Table 3, entries 2–3). When the graft density was further increased to 18.0%, however, the nanosphere became larger (212.6 nm), accompanied by a drop of the density (Figure 3D; Table 3, entry 4), suggesting that more PLA side-chains aggregated into one nanosphere. Under the same graft density (8.6%), AFM phase images of copolymers with short grafts such as PS_8.6_–*g*–PLA_5_ (228/(100 μm^2^)) and PS_8.6_–*g*–PLA_10_ (239/(100 μm^2^)) showed a similar density of nanospheres (Figure 4A,B; Table 3, entries 2 and 5), while the long PLA repeat unit such as PS_8.6_–g–PLA_20_ led to a reduced nanosphere density of 169/(100 μm^2^) (Figure 4C; Table 3, entry 6), which might be attributed to the serious chain entanglement. After etching the PLA phase by NaOH/methanol solution, the corresponding nanoporous films were confirmed by TEM (Figure S3, Supplementary Material).

**Table 3:** The average particle size and number of the PS–*g*–PLA with different graft densities and lengths after thermal annealing. ^a^

Entry	PS–*g*–PLA	Average particle size ^b^ (nm)	Average particle number ^b^ (100 μm^2^)
1	PS_4.3_–*g*–PLA_10_	130.1	179
2	PS_8.6_–*g*–PLA_10_	158.2	239
3	PS_12.5_–*g*–PLA_10_	152.4	293
4	PS_18.0_–*g*–PLA_10_	212.6	158
5	PS_8.6_–*g*–PLA_5_	133.5	228
6	PS_8.6_–*g*–PLA_20_	224.2	169

^a^ Conditions: thermal annealing temperature: 115 °C, time: 4 h; ^b^ The average particle size and number of the PS–*g*–PLA were measured by Nano Measurer 1.2.

## 4. Conclusions

This work, for the first time, demonstrated that even in the presence of PS–OH as the protic alcohol, the *immortal* ROP of LA could also proceed, in a controllable manner, to generate a series of PS–*g*–PLA copolymers with different graft densities and graft lengths through controlling the feeding of LA and the content of the hydroxyl group within PS–OH. After thermal annealing, the PLA domain aggregated to nanospheres among the PS continuum. Under the same PLA graft length, the density of the nanosphere increased from 179 to 293/(100 μm^2^) with the graft density of PLA increasing in the range of 4.3% and 12.5%, which dropped at further increasing of the graft density due to more PLA participating in the aggregation to form a large nanosphere. When the graft density was kept constant, the short graft chain length seemed affected the diameter of the nanosphere more obviously, while the serious chain entanglement caused by long side-chains reduced the nanosphere density. Furthermore, the chemical etching of the PLA component resulted in a large number of hydroxyl functionalizations within the porous membrane. This functionality could be the “reactive handles” to achieve membranes of various applications.

## Supplementary Materials

The following are available online at www.mdpi.com/2073-4360/8/1/17/s1.

## References

[1] Hsu Y.H., Chiang W.H., Chen M.C., Chern C.S., Chiu H.C. (2006). Effects of SDS on the thermo- and pH-sensitive structural changes of the poly(acrylic acid)-based copolymer containing both poly(*N*-isopropylacrylamide) and monomethoxy poly(ethylene glycol) grafts in water. Langmuir.

[2] Lee H.J., Yang S.R., An E.J., Kim J.D. (2006). Biodegradable polymersomes from poly(2-hydroxyethyl aspartamide) grafted with lactic acid oligomers in aqueous solution. Macromolecules.

[3] Bougard F., Giacomelli C., Mespouille L., Borsali R., Dubois P., Lazzaroni R. (2008). Influence of the macromolecular architecture on the self-assembly of amphiphilic copolymers based on poly(*N*,*N*-dimethylamino-2-ethyl methacrylate) and poly(epsilon-caprolactone). Langmuir.

[4] Zhang J.X., Qiu L.Y., Zhu K.J. (2005). Solvent controlled multi-morphological self-assembly of amphiphilic graft copolymers. Macromol. Rapid Commun..

[5] Balomenou I., Bokias G. (2005). Water-soluble complexes between cationic surfactants and comb-type copolymers consisting of an anionic backbone and hydrophilic nonionic poly(*N*,*N*-dimethylacrylamide) side chains. Langmuir.

[6] Wan S., Jiang M., Zhang G.Z. (2007). Dual temperature- and pH-dependent self-assembly of cellulose-based copolymer with a pair of complementary grafts. Macromolecules.

[7] Petit L., Karakasyan C., Pantoustier N., Hourdet D. (2007). Synthesis of graft polyacrylamide with responsive self-assembling properties in aqueous media. Polymer.

[8] Zhang X.H., Shen Z., Feng C., Yang D., Li Y.G., Hu J.H., Lu G.L., Huang X.Y. (2009). PMHDO–*g*–PEG double-bond-based amphiphilic graft copolymer: Synthesis and diverse self-assembled nanostructures. Macromolecules.

[9] Heroguez V., Gnanou Y., Fontanille M. (1997). Novel amphiphilic architectures by ring-opening metathesis polymerization of macromonomers. Macromolecules.

[10] Xia Y., Olsen B.D., Kornfield J.A., Grubbs R.H. (2009). Efficient synthesis of narrowly dispersed brush copolymers and study of their assemblies: The importance of side-chain arrangement. J. Am. Chem. Soc..

[11] Stephan T., Muth S., Schmidt M. (2002). Shape changes of statistical copolymacromonomers: From wormlike cylinders to horseshoe- and meanderlike structures. Macromolecules.

[12] Ishizu K., Sawada N., Satoh J., Sogabe A. (2003). Architecture and surfactant behavior of amphiphilic prototype copolymer brushes. J. Mater. Sci. Lett..

[13] Zhang Y., Li X.K., Deng G.H., Chen Y.M. (2006). Novel hybrid polymer brushes with alternating dendritic wedges and linear side chains. Macromol. Chem. Phys..

[14] Wu D.X., Yang Y.F., Cheng X.H., Liu L., Tian J., Zhao H.Y. (2006). Mixed molecular brushes with PLLA and PS side chains prepared by AGET ATRP and ring-opening polymerization. Macromolecules.

[15] Xie M.R., Dang J.Y., Han H.J., Wang W.Z., Liu J.W., He X.H., Zhang Y.Q. (2008). Well-defined brush copolymers with high grafting density of amphiphilic side chains by combination of ROP, ROMP, and ATRP. Macromolecules.

[16] Fu Q., Liu C., Lin W.C., Huang J.L. (2008). One-pot synthesis of heterograft copolymers via “graft onto” by atom transfer nitroxide radical coupling chemistry. J. Polym. Sci. A.

[17] Feng C., Li Y.J., Yang D., Hu J.H., Zhang X.H., Huang X.Y. (2011). Well-defined graft copolymers: From controlled synthesis to multipurpose applications. Chem. Soc. Rev..

[18] Huang K., Rzayev J. (2009). Well-defined organic nanotubes from multicomponent bottlebrush copolymers. J. Am. Chem. Soc..

[19] Asano S., Aida T., Inoue S. (1985). Immortal polymerization—Polymerization of epoxide catalyzed by an aluminum porphyrin alcohol system. J. Chem. Soc. Chem..

[20] Aida T., Inoue S. (1996). Metalloporphyrins as initiators for living and immortal polymerizations. Acc. Chem. Res..

[21] Inoue S. (2000). Immortal polymerization: The outset, development, and application. J. Polym. Sci. A.

[22] Shueh M.L., Wang Y.S., Huang B.H., Kuo C.Y., Lin C.C. (2004). Reactions of 2,2′-methylenebis(4-chloro-6-isopropyl-3-methylphenol) and 2,2′-ethylidenebis(4,6-di-*tert*-butylphenol) with Mg^n^Bu_2_: Efficient catalysts for ring-opening polymerization of epsilon-caprolactone and l-lactide. Macromolecules.

[23] Amgoune A., Thomas C.M., Carpentier J.F. (2007). Yttrium complexes as catalysts for living and immortal polymerization of lactide to highly heterotactic PLA. Macromol. Rapid Commun..

[24] Sarazin Y., Liu B., Roisnel T., Maron L., Carpentier J.F. (2011). Discrete, solvent-free alkaline-earth metal cations: Metal center dot center dot center dot fluorine interactions and ROP catalytic activity. J. Am. Chem. Soc..

[25] Wang Y., Zhao W., Liu X., Cui D., Chen E.Y.X. (2012). Ligand-free magnesium catalyst system: immortal polymerization of l-Lactide with high catalyst efficiency and structure of active intermediates. Macromolecules.

[26] Zhao W., Wang Y., Liu X., Cui D. (2012). Facile synthesis of pendant- and α,γ-chain-end-functionalized polycarbonates via immortal polymerization by using a salan lutetium alkyl precursor. Chem. Commun..

[27] Zhao W., Wang Y., Liu X., Chen X., Cui D., Chen E.Y. (2012). Protic compound mediated living cross-chain-transfer polymerization of rac-lactide: Synthesis of isotactic (crystalline)-heterotactic (amorphous) stereomultiblock polylactide. Chem. Commun..

[28] Zhao W., Li C.Y., Liu B., Wang X., Li P., Wang Y., Wu C.J., Yao C.G., Tang T., Liu X.L. (2014). A new strategy to access polymers with aggregation-induced emission characteristics. Macromolecules.

[29] Zhao W., Cui D.M., Liu X.L., Chen X.S. (2010). Facile synthesis of hydroxyl-ended, highly stereoregular, star-shaped poly(lactide) from immortal ROP of *rac*-Lactide and kinetics study. Macromolecules.

[30] Liu N., Yao C., Lin F., Liu B., Cui D. (2015). An intensification and integration process of preparing thermal stable polylactide end-capped by phosphate ester. Polymer.

[31] Bigot Y.L., Delmas M., Gaset A. (2006). A simplified wittig synthesis using solid/liquid transfer processes IV—Synthesis of symmetrical and asymmetrical mono-and di-olefins from terephtalic aldehyde. Synth. Commun..

[32] Odian G. (2004). Principles of Polymerization.

[33] Li S.M. (1999). Hydrolytic degradation characteristics of aliphatic polyesters derived from Lactic and Glycolic acids. J. Biomed. Mater. Res..

[34] Mark J.E. (2006). Some novel polymeric nanocomposites. Acc. Chem. Res..

[35] Olayo-Valles R., Guo S.W., Lund M.S., Leighton C., Hillmyer M.A. (2005). Perpendicular domain orientation in thin films of polystyrene—Polylactide diblock copolymers. Macromolecules.

