# Supplementary material for: Immortal Ring-Opening Polymerization of rac-Lactide Using Polymeric Alcohol as Initiator to Prepare Graft Copolymer
Source: Polymers (Basel). 2016 Jan 15;8(1):17. doi: 10.3390/polym8010017 (PMC6432534; doi:10.3390/polym8010017)
Supplement: Supplementary file 1 [file polymers-08-00017-s001.pdf]

# Supplementary Materials: *Immortal* Ring-Opening Polymerization of *rac*-Lactide Using Polymeric Alcohol as Initiator to Prepare Graft Copolymer

**Na Liu, Bo Liu, Changguang Yao and Dongmei Cui**

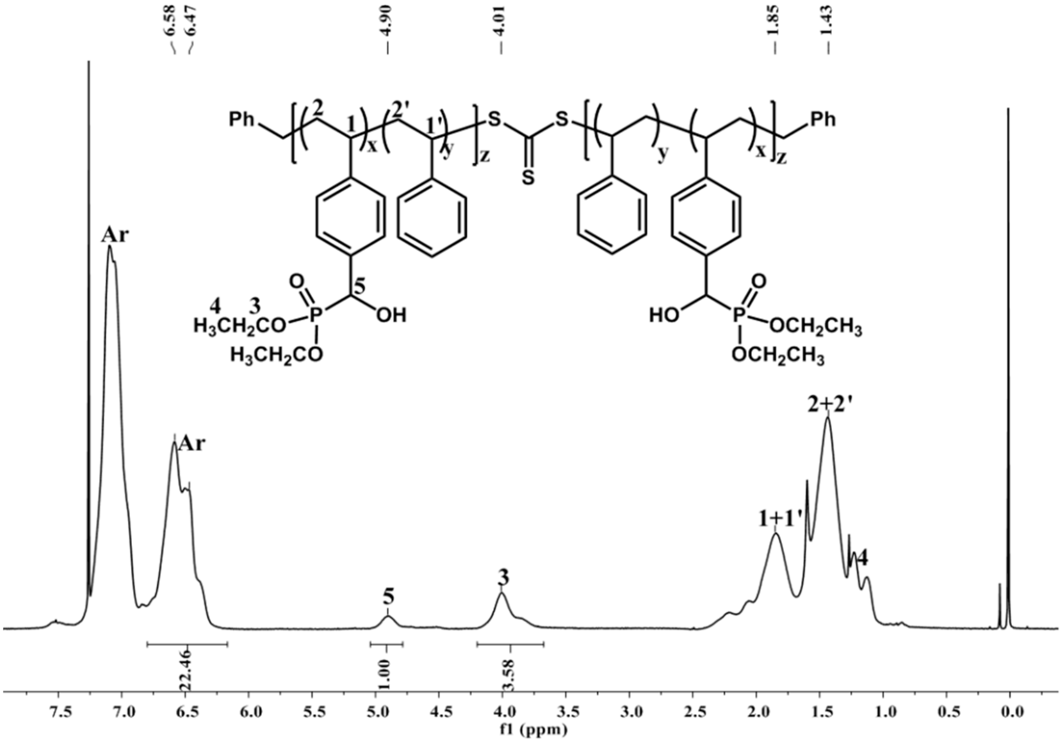


**Figure S1.** $^1$H NMR spectrum (400 MHz, $CDCl_3$, 298 K) of the PS–OH ($M_n$ = 8800 g·mol$^{-1}$, PDI = 1.14).

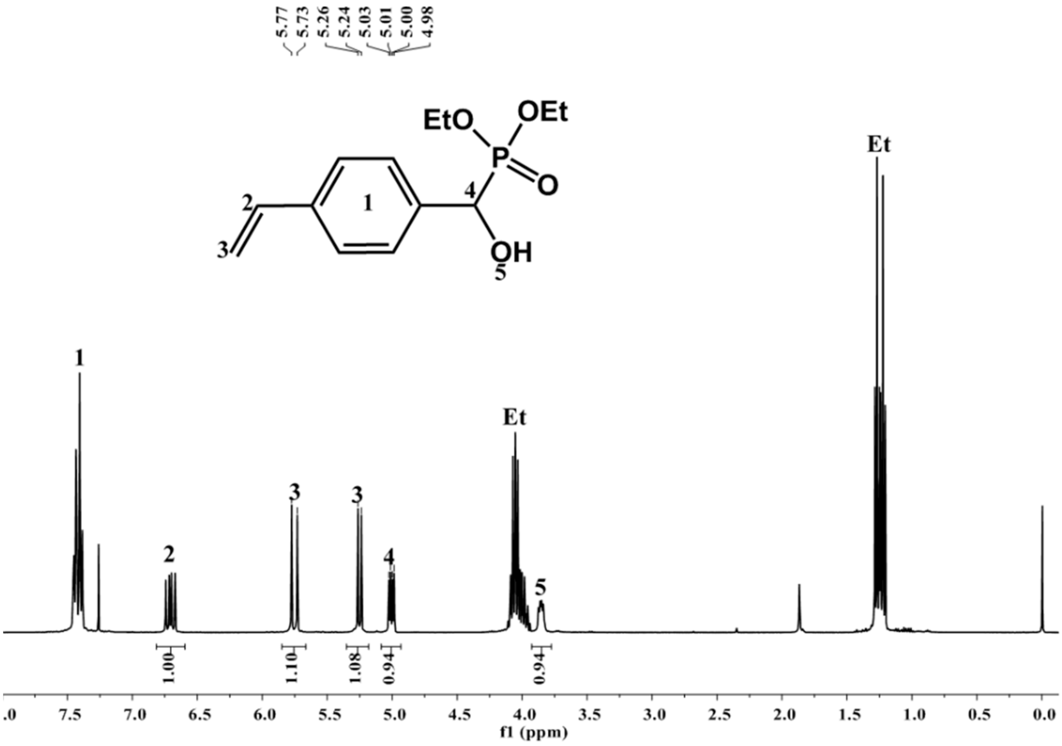


**Figure S2.** $^1$H NMR spectrum (400 MHz, $CDCl_3$, 298 K) of the St–OH.

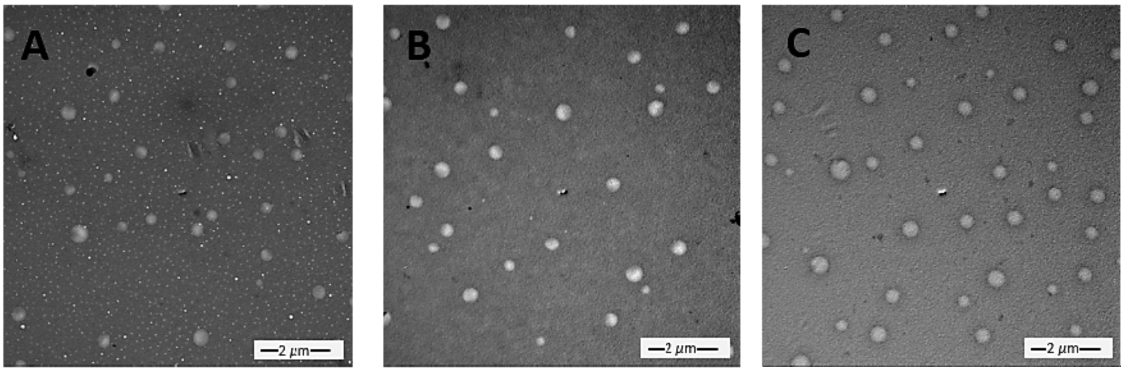


**Figure S3.** TEM images of the PS-*g*-PLA with different graft lengths after etching: (**A**) $PS_{8.6}$–*g*–$PLA_{5}$ (Table 2, entry 5); (**B**) $PS_{8.6}$–*g*–$PLA_{10}$ (Table 2, entry 2); (**C**) $PS_{8.6}$–*g*–$PLA_{20}$ (Table 2, entry 6). Conditions: accelerating voltage: 100 kV; amplification factor: 15,000.

© 2016 by the authors; licensee MDPI, Basel, Switzerland. This article is an open access article distributed under the terms and conditions of the Creative Commons by Attribution (CC-BY) license (http://creativecommons.org/licenses/by/4.0/).
